# Impact of sleep deprivation on creatine metabolism biomarkers in healthy young males

**DOI:** 10.1080/15502783.2025.2533643

**Published:** 2025-07-17

**Authors:** Nikola Todorovic, David Nedeljkovic, Jovana Panic, Sergej M. Ostojic

**Affiliations:** a University of Novi Sad, Applied Bioenergetics Lab, Faculty of Sport and Physical Education, Novi Sad, Serbia; b University of Novi Sad, Faculty of Sciences, Novi Sad, Serbia; c University of Pécs, Faculty of Health Sciences, Pécs, Hungary; d University of Agder, Department of Nutrition and Public Health, Kristiansand, Norway

**Keywords:** Inadequate sleep, creatine, biomarkers, metabolism

## Abstract

**Background:**

Creatine is a crucial component of cellular energy metabolism, facilitating ATP regeneration to support high energy demands. While its therapeutic potential, particularly in neuroprotection, is well-recognized, its response to stress conditions such as sleep deprivation remains insufficiently investigated. This study examines the effects of sleep deprivation on creatine metabolism in healthy young males.

**Methods:**

Sixteen healthy young male participants (age: 25.3 ± 4.7 years; body mass: 81.6 ± 8.8 kg) were recruited to assess the impact of 24-hour sleep deprivation on biomarkers of creatine metabolism. Primary outcomes included serum levels of creatine, guanidinoacetic acid (GAA), and creatinine, measured at baseline and after 24 hours.

**Results:**

Sleep deprivation significantly increased serum creatine levels, rising from 48.63 ± 27.05 μmol/L at baseline to 63.84 ± 23.83 μmol/L after 24 hours (95% CI: −29.14 to −1.27; *P* = 0.03), with a moderate to large effect size (Cohen’s *d* = 0.59). Serum GAA levels showed a non-significant increase, from 3.27 ± 0.60 μmol/L to 3.47 ± 0.64 μmol/L (95% CI: −0.58 to 0.16; *P* = 0.26; Cohen’s *d* = 0.32). Serum creatinine levels remained unchanged, with no significant difference between baseline (100.57 ± 9.19 μmol/L) and follow-up (106.10 ± 27.90 μmol/L; 95% CI: −21.24 to 10.20; *P* = 0.46; Cohen’s d = 0.26).

**Conclusion:**

Sleep deprivation alters creatine metabolism biomarkers, indicating shifts in metabolic pathways. Further research is necessary to validate and expand these findings, potentially elucidating the role of creatine in stress-related conditions.

